# Diagnostic Ambiguity of Encephalopathy in Advanced Kidney Disease: Multisystem Overlap in a Patient With Autosomal Dominant Polycystic Kidney Disease (ADPKD), Heart Failure, and Stiff Person Syndrome

**DOI:** 10.7759/cureus.106269

**Published:** 2026-04-01

**Authors:** Christopher M Ahmad, Rae-Anne Kastle, Ishita Agarwal, Samantha Elliott-Ventura, Muhammad Farooq

**Affiliations:** 1 College of Medicine, Kansas City University, Joplin, USA; 2 Internal Medicine, Mercy Hospital Joplin, Joplin, USA

**Keywords:** adpkd, diagnostic uncertainty, end-stage renal disease, hfpef, renal replacement therapy planning, stiff person syndrome, uremic encephalopathy

## Abstract

Altered mental status (AMS) in advanced chronic kidney disease (CKD) frequently reflects overlapping metabolic, cardiopulmonary, and neurologic contributors rather than a single unifying diagnosis. We present a 59-year-old woman with autosomal dominant polycystic kidney disease (ADPKD) progressing to end-stage renal disease (ESRD), congestive heart failure (CHF) with preserved ejection fraction (HFpEF), and stiff person syndrome (SPS) who developed recurrent encephalopathy accompanied by equivocal pulmonary findings and elevated cardiac troponin without electrocardiographic evidence of acute ischemia. Her hospital course was marked by severe renal dysfunction, metabolic acidosis, anemia, and intermittent electrolyte abnormalities. Management required iterative reassessment rather than reliance on a single explanatory diagnosis, included time-variable use of antibiotics and diuretics, continued chronic cardiometabolic therapies, and preparation for renal replacement therapy. This case highlights three practical teaching points: encephalopathy in ESRD is often multifactorial; troponin elevation in advanced kidney disease requires careful clinical correlation to avoid misclassification as acute coronary syndrome; and distinguishing pneumonia from pulmonary edema in volume-sensitive patients may necessitate repeated reassessment over time.

## Introduction

Altered mental status (AMS) in advanced chronic kidney disease (CKD) is a common presentation, but it rarely has a single explanation. Metabolic derangements, uremia, hypoxemia/hypercapnia, anemia, infection, and cardiopulmonary instability can coexist and fluctuate over short time horizons, making the clinical picture vulnerable to premature closure. Bugnicourt et al. described two potential hypotheses leading to brain damage in patients with CKD: the vascular hypothesis and neurodegenerative hypothesis [1]. Both hypotheses encompass non-traditional risk factors (e.g., chronic inflammation and oxidative stress) and uremic toxins; however, the vascular hypothesis also includes traditional risk factors (e.g., ageing, hypertension, and diabetes mellitus) [1]. This further indicates the complexity of neurological complications in patients with CKD.

Another complication of CKD is the relationship to cardiovascular diseases. Thirty percent of patients with CKD have self-reported a history of cardiovascular disease [2]. A total of 47-57% of patients with CKD are on an angiotensin-converting enzyme (ACE) inhibitor [3]. Additionally, CKD is among the most common causes of troponin elevation, a marker of cardiac damage [2]. Interpreting troponin levels can be challenging in this setting, where baseline abnormalities and competing etiologies are common [2]. Lastly, CKD increases the prevalence of pulmonary comorbidities, regardless of disease stage, by altering fluid homeostasis [4,5]. This leads to fluid overload in the lungs, resulting in opacities. These opacities are mostly bilateral but, in rare instances, can be unilateral and may form a consolidation [5].

We present a patient with autosomal dominant polycystic kidney disease (ADPKD) progressing to end-stage renal disease (ESRD), congestive heart failure (CHF) with preserved ejection fraction (HFpEF), and stiff person syndrome (SPS) with pulmonary findings that alternated between suspected infection and volume overload, who developed recurrent encephalopathy. This report is deliberately framed around bedside decision-making, including approaching encephalopathy as multifactorial in ESRD [1], interpreting troponin elevation in advanced kidney disease without anchoring on acute coronary syndrome [2], and adjudicating pneumonia versus cardiogenic pulmonary edema when both remain plausible [5]. SPS is included as a baseline neurologic comorbidity that may complicate assessment and functional recovery rather than as a singular causal explanation for encephalopathy. This case illustrates how diagnostic momentum in medically complex patients can shift over hours to days, requiring clinicians to repeatedly re-evaluate working diagnoses rather than pursue a single unifying explanation.

## Case presentation

A 59-year-old woman with a complex medical history, including ADPKD, CHF with HFpEF, and SPS, presented with recurrent episodes of AMS, generalized weakness, and progressive functional decline. Her surgical history was notable for a prior cerebral aneurysm clipping and a recently created arteriovenous (AV) fistula (April 2025) in anticipation of ESRD. No focal neurologic deficits, cranial nerve abnormalities, cerebellar signs, or gait instability were observed. No symptoms of nausea or vomiting were reported.

Physical exam and laboratory findings

**Figure 1:**
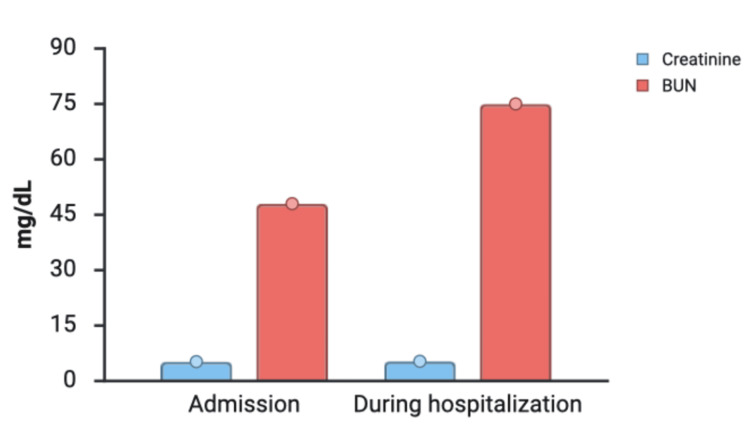
Trends of creatinine and blood urea nitrogen (BUN) at admission and during the hospital stay. mg/dL: milligrams per deciliter Source: This is an original, author-created graph using BioRender (BioRender.com, Toronto, ON, Canada) without the use of artificial intelligence (AI).

Upon admission, the patient was somnolent but arousable to verbal stimuli. Initial vital signs were stable, except for elevated blood pressure at 155/93. Laboratory evaluation confirmed severe renal dysfunction (stage 5 CKD) with a creatinine of 5.33 mg/dL, blood urea nitrogen (BUN) of 75 mg/dL, and an estimated glomerular filtration rate (eGFR) <10 mL/min/1.73 m^2^, shown in Figure 1. Metabolic derangements included a bicarbonate of 22 mmol/L, an anion gap of 18, and hyperphosphatemia. Additional laboratory findings and their clinical relevance are noted in Table 1. Notably, troponin T was elevated at 66 ng/L (baseline unknown), while electrocardiography (ECG) showed no acute ST-T wave changes. An arterial blood gas (ABG) on room air demonstrated a primary respiratory acidosis (pH 7.28, pCO_2_ 49 mmHg) and significant hypoxemia (oxygen saturation 37% (normal 40-70%)).

**Table 1:** Key laboratory findings on admission and during hospitalization. ESRD: end-stage renal disease; BUN: blood urea nitrogen; GFR: glomerular filtration rate; CKD: chronic kidney disease; ECG: electrocardiogram; pCO_2_: partial pressure of carbon dioxide; O_2_: oxygen; mg: milligrams; dL: deciliter; mL: milliliters; L: liters; min: minute; mmol: millimole; mmHg: millimeter of mercury; g: grams; ng: nanograms; µmol: micromole; pH: potential of hydrogen

Laboratory Parameter	Observed Values	Reference Ranges	Clinical Relevance
Creatinine	5.23-5.33 mg/dL	0.51-0.95 mg/dL	Advanced renal dysfunction consistent with ESRD
BUN	48-75 mg/dL	6-20 mg/dL	Uremic burden contributing to encephalopathy
Estimated GFR	<10 mL/min/1.73 m^2^	≥60 mL/min/1.73 m^2^	ESRD
Phosphorus	5.5 mg/dL	2.5-4.5 mg/dL	Hyperphosphatemia in advanced CKD
Potassium	Up to 5.3 mmol/L	3.5-5.1 mmol/L	Intermittent hyperkalemia
Bicarbonate (CO_2_)	22 mmol/L	22-29 mmol/L	Metabolic acidosis
Anion gap	14-18	4-13	Elevated anion gap metabolic acidosis
Arterial pH	7.28	7.32-7.42	Acidemia
Arterial pCO_2_	49 mmHg	41-51 mmHg	Respiratory contribution to acid–base disturbance
Arterial O_2_ saturation	37%	40-70%	Hypoxemia on arterial blood gas
Hemoglobin	9.6-10.4 g/dL	12.5-16 g/dL	Anemia of chronic disease
Hematocrit	26-31%	37-47	Reduced oxygen-carrying capacity
Troponin	66 ng/L	<10 ng/L	Elevated without ECG evidence of acute ischemia
Ammonia	<10 µmol/L	11-51 µmol/L	Hepatic encephalopathy unlikely

Imaging and diagnostics

**Figure 2:**
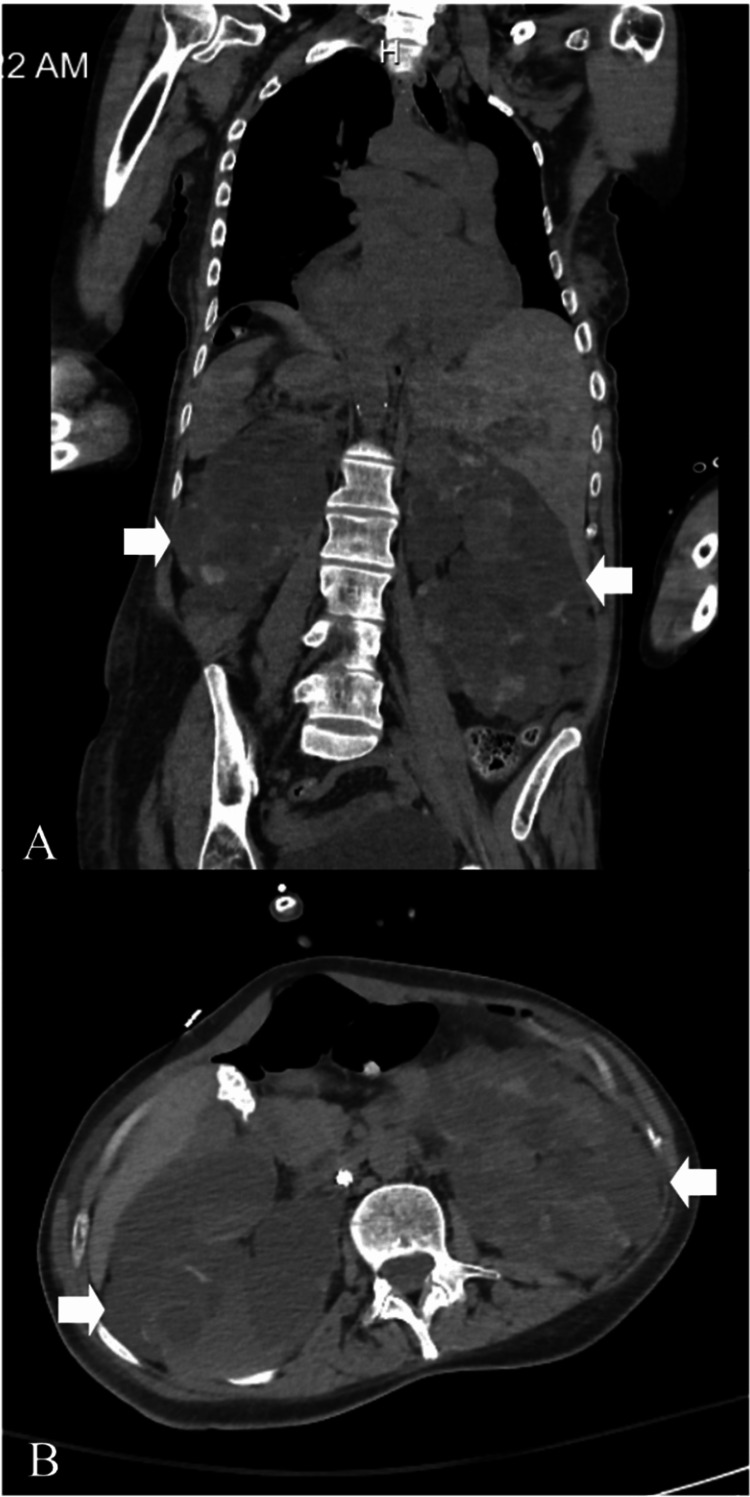
Contrast-enhanced computed tomography demonstrating advanced autosomal dominant polycystic kidney disease (ADPKD) (white arrows). (A) Coronal view demonstrating markedly enlarged bilateral kidneys replaced by innumerable fluid-attenuation cysts (white arrows). (B) Axial view demonstrating diffuse cystic replacement of renal parenchyma with loss of normal corticomedullary architecture (white arrows). These findings are consistent with long-standing ADPKD and correlate with the patient’s progression to end-stage renal disease (ESRD).

**Figure 3:**
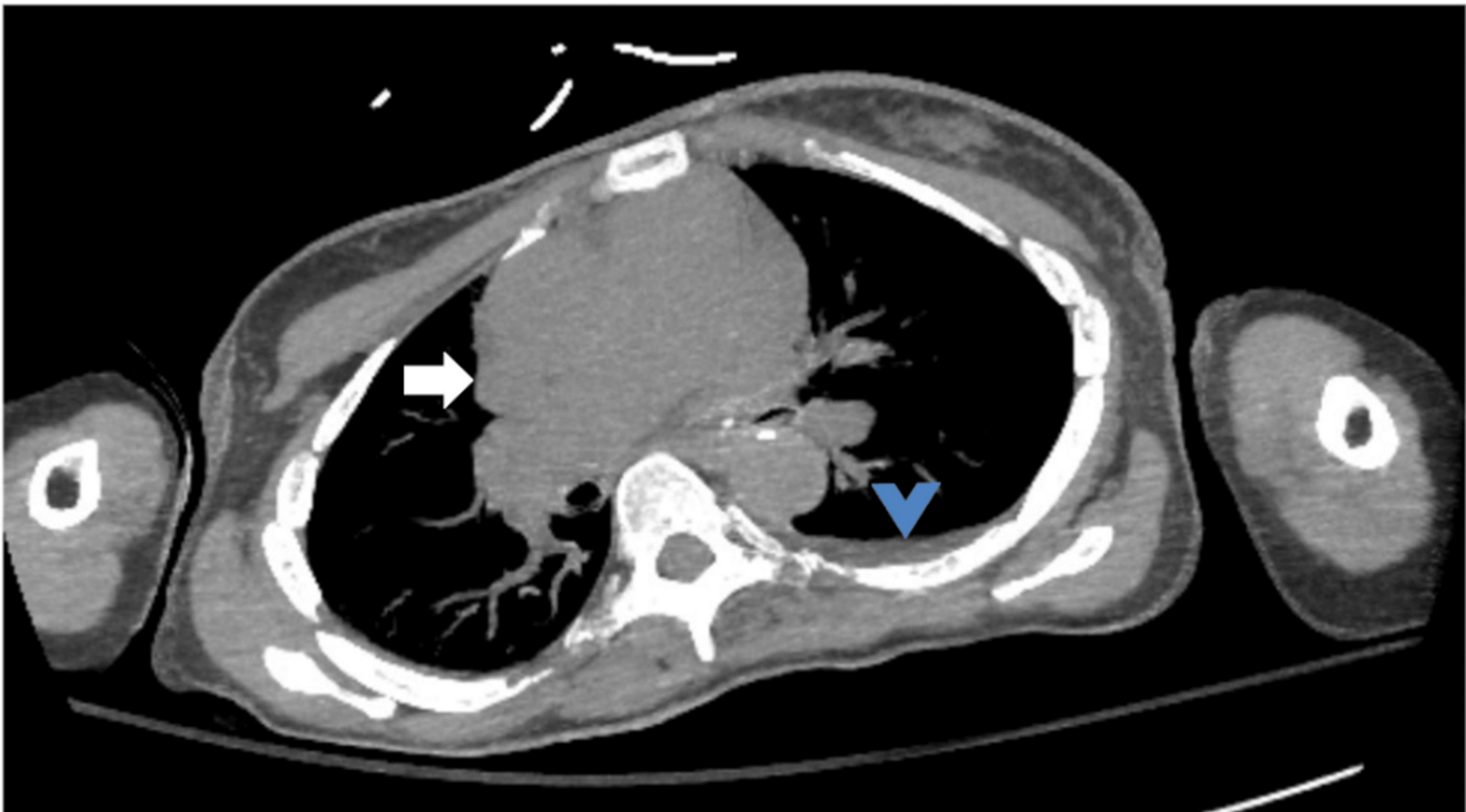
Contrast-enhanced computed tomography of the chest demonstrating cardiomegaly (white arrow) and small left pleural effusion (blue arrowhead). No focal lobar consolidation is identified.

**Figure 4:**
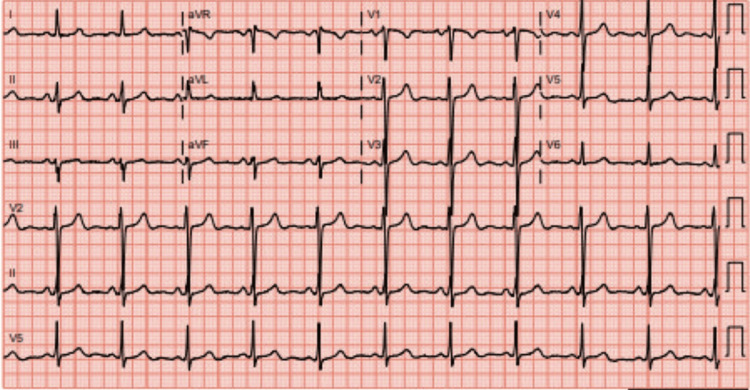
Electrocardiogram (EKG) demonstrating normal sinus rhythm with possible left atrial enlargement and no signs of ischemia.

Neuroimaging, a non-contrast computed tomography (CT) of the head, revealed chronic postsurgical changes from her aneurysm clipping and left temporal encephalomalacia, but no acute intracranial pathology. The CT scan of the chest, abdomen, and pelvis showed bilateral polycystic kidney disease (Figure 2), a small left pleural effusion (Figure 3), and cardiomegaly with a small pericardial effusion (Figure 3). The electrocardiogram (EKG) showed a normal sinus rhythm with possible left atrial enlargement, but no signs of ischemia (Figure 4). Transthoracic echocardiography (TTE) confirmed concentric left ventricular hypertrophy with a preserved ejection fraction (55-60%) and elevated right ventricular systolic pressure (45-50 mmHg), shown in Table 2.

**Table 2:** Imaging and cardiac testing summary. CT: computed tomography; LV: left ventricle; EF: ejection fraction; RVSP: right ventricular systolic pressure; ADPKD: autosomal-dominant polycystic kidney disease; EKG: electrocardiogram; ACS: acute coronary syndrome

Study	Key Findings	Interpretation
CT brain (non-contrast)	Prior aneurysm clipping; left temporal encephalomalacia; small vessel ischemic disease; no acute hemorrhage	Chronic neurologic substrate without acute intracranial pathology
CT abdomen/pelvis (Figure 2)	Innumerable bilateral renal cysts	Findings consistent with ADPKD
CT chest (Figure 3)	Small left pleural effusion; mild pericardial effusion	Findings consistent with cardiopulmonary involvement
Transthoracic echocardiogram	Concentric LV hypertrophy; EF 55-60%; mild mitral and tricuspid regurgitation; RVSP 45-50 mmHg	Heart failure with preserved ejection fraction and elevated pulmonary pressures
EKG (Figure 4)	No acute ischemic changes	Troponin elevation is unlikely due to ACS

Hospital course

The clinical course was defined by a diagnostic tug-of-war between infectious and cardiogenic etiologies, as outlined in Table 3. At presentation, pulmonary opacities and hypoxemia prompted initiation of broad-spectrum antibiotics for presumed pneumonia. However, the absence of definitive infectious markers and persistence of radiographic findings led to reconsideration of volume overload as a primary contributor. Diuretic therapy was subsequently initiated, with partial improvement in respiratory status. Despite these interventions, the patient’s mental status fluctuated, supporting a multifactorial etiology including uremic and metabolic contributors. Her SPS was managed with her home regimen of baclofen and diazepam, though these centrally acting agents were briefly held to assess their contribution to AMS. Despite multidisciplinary management, the patient experienced a slow functional recovery and was discharged with plans for imminent hemodialysis initiation via her maturing fistula.

**Table 3:** Detailed timeline of hospital course and clinical reassessment. This progression underscores how no single intervention produced complete resolution, reinforcing the interpretation of encephalopathy as a composite of interacting metabolic, cardiopulmonary, and neurologic factors. HFpEF: heart failure with preserved ejection fraction; ESRD: end-stage renal disease; AMS: altered mental status; AV: arteriovenous; ABG: arterial blood gas; pH: potential of hydrogen; CT: computed tomography; pCO_2_: partial pressure of carbon dioxide; ECG: electrocardiogram; Cr: creatinine; mg/dL: milligrams per deciliter; BUN: blood urea nitrogen; eGFR: estimated glomerular filtration rate

Hospital Day/Phase	Key Clinical Findings	Objective Data	Working Diagnosis	Interventions	Clinical Response
Day 0 (Admission)	AMS, hypoxemia, pulmonary opacities, pleural effusion	ABG: pH 7.28, pCO_2_ 49 mmHg, low O_2_ saturation; CT chest: pleural effusion without focal consolidation; elevated troponin (66 ng/L) without ECG changes	Pneumonia vs. volume overload; multifactorial encephalopathy suspected	Broad-spectrum antibiotics initiated; oxygen support	Minimal improvement in mental status; persistent hypoxemia
Day 1-2 (Early Course)	Persistent AMS; no fever; stable imaging; no clear infectious source	No leukocytosis; imaging unchanged; renal dysfunction persists (Cr >5 mg/dL, BUN rising)	Infection less supported; volume overload and uremia increasingly favored	Continued antibiotics initially; diuretics initiated for volume management	Mild improvement in oxygenation; AMS unchanged
Day 3-4 (Reassessment Phase)	Fluctuating mental status; persistent metabolic abnormalities	Ongoing metabolic acidosis, hyperphosphatemia, anemia; no new infectious markers	Uremic/metabolic encephalopathy prioritized; pneumonia less likely	Antibiotics de-escalated; diuretics optimized; medication review (baclofen/diazepam briefly held)	Partial improvement in respiratory status; AMS persists but fluctuates
Mid-late Hospital Course	Continued AMS with gradual stabilization; no new focal findings	Persistent ESRD-level labs (eGFR <10); stable cardiac findings (no ECG changes); no progression of pulmonary findings	Multifactorial encephalopathy confirmed (uremia + metabolic + cardiopulmonary contributors)	Continued diuresis; supportive care; planning for renal replacement therapy	Gradual improvement in mental status but not baseline
Discharge Phase	Improved alertness but residual cognitive slowing	Stable but abnormal renal function; no acute cardiopulmonary deterioration	Chronic multifactorial encephalopathy in ESRD	Home medications resumed; outpatient dialysis planning via AV fistula	Clinically stable for discharge with follow-up for dialysis initiation

Discharge and follow-up

At discharge, the patient demonstrated improved alertness compared to admission, though mild cognitive slowing persisted and had not fully returned to her baseline. Metabolic parameters remained consistent with ESRD without evidence of new acute derangements. She was hemodynamically stable on room air with improved respiratory status following diuresis.

Given persistent renal dysfunction and recurrent metabolic instability, she was discharged with closed outpatient nephrology follow-up and plans for imminent initiation of hemodialysis via her recently created AV fistula. Ongoing management was expected to focus on the coordination of renal placement therapy and optimization of her cardiopulmonary comorbidities.

## Discussion

This case illustrates a common yet challenging reality in the care of patients with ESRD: diagnostic ambiguity arising from overlapping, plausible etiologies rather than a single rare diagnosis. The patient’s recurrent encephalopathy occurred in the setting of severe renal dysfunction, metabolic acidosis, anemia, intermittent electrolyte abnormalities, abnormal gas exchange, and cardiopulmonary instability, each of which can independently contribute to AMS [6]. The principal clinical risk in such cases is not failure to identify an exotic diagnosis, but premature closure on a single explanation despite evolving data.

Encephalopathy in ESRD as a multifactorial syndrome

**Figure 5:**
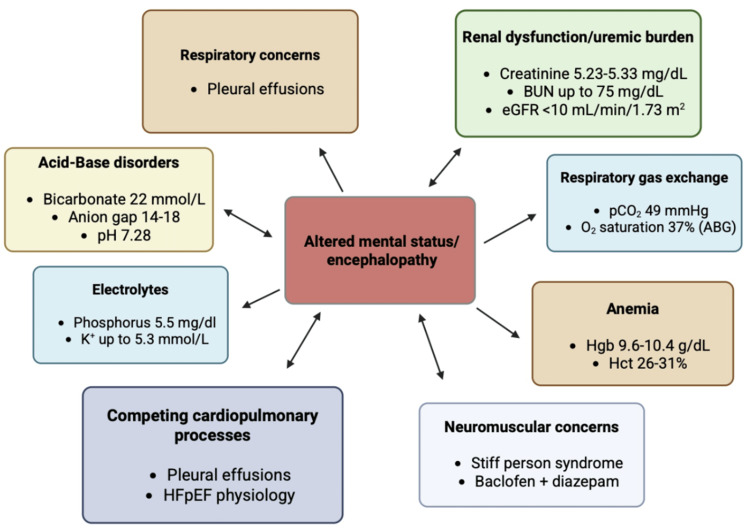
Schematic illustration of multifactorial contributors to encephalopathy in a patient with advanced chronic kidney disease and multisystem comorbidity. mg: milligrams; dL: deciliter; BUN: blood urea nitrogen; eGFR: estimated glomerular filtration rate; mL: milliliter; min: minutes; m^2^: meters squared; pCO_2_: partial pressure of carbon dioxide; O_2_: oxygen; ABG: arterial blood gas; Hgb: hemoglobin; Hct: hematocrit; HFpEF: heart failure with preserved ejection fraction; mmol: milimoles; L: liter; pH: potential of hydrogen Source: This is an original, author-created graph using BioRender (BioRender.com, Toronto, ON, Canada) without the use of artificial intelligence (AI).

In advanced kidney disease, encephalopathy is best approached as a multifactorial syndrome rather than a discrete diagnosis, shown in Figure 5 [7]. Uremic physiology, acid-base disturbance, electrolyte shifts, hypoxemia or hypercapnia, infection, medication effects, and diminished physiologic reserve frequently coexist and interact [8]. In this patient, objective abnormalities, including metabolic acidosis, hyperphosphatemia, intermittent hyperkalemia, anemia, and abnormal ABG parameters, supported a multi-driver framework. The low ammonia level helped deprioritize hepatic encephalopathy; however, the patient’s encephalopathy persisted despite sequential targeted interventions, including empiric antibiotic therapy initiated for suspected pneumonia and later diuretic therapy for presumed volume overload, with only partial improvement in respiratory status and continued fluctuation in mental status. This pattern reinforced the need for ongoing reassessment rather than reliance on a single diagnostic framework [9].

Baseline neurologic disease further complicated the assessment. While SPS was not considered a primary cause of encephalopathy, chronic neurologic impairment and use of centrally acting medications may reduce neurologic reserve and obscure subtle changes in mental status [10]. This underscores the importance of contextual interpretation of neurologic findings in patients with preexisting neurologic disorders.

Troponin elevation in advanced kidney disease

**Figure 6:**
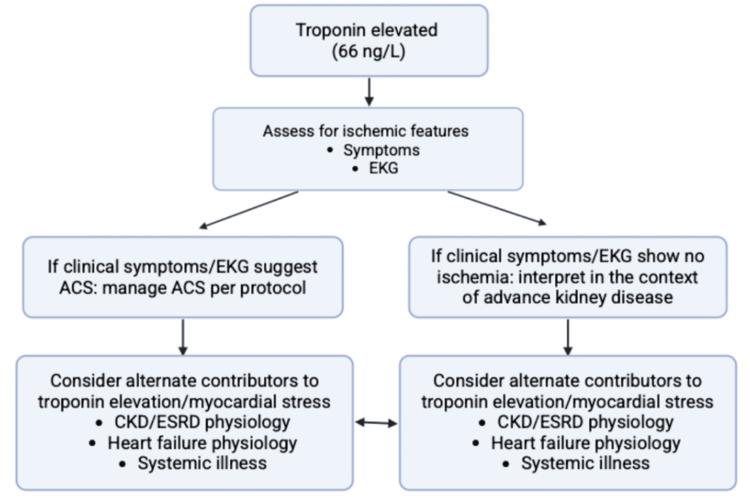
Flow diagram illustrating a structured approach to interpreting troponin elevation in patients with advanced CKD. ng: nanograms; L: liter; EKG: electrocardiogram; ACS: acute coronary syndrome; CKD: chronic kidney disease; ESRD: end-stage renal disease Source: This is an original, author-created graph using BioRender (BioRender.com, Toronto, ON, Canada) without the use of artificial intelligence (AI).

The elevated troponin level in the absence of ischemic electrocardiographic changes exemplifies a common diagnostic dilemma in advanced CKD and ESRD, shown in Figure 6. Troponin elevation in this population may reflect chronic myocardial strain, structural heart disease, or systemic illness rather than acute coronary syndrome [11]. In this case, the absence of ischemic ECG findings (Figure 4) and the broader context of multisystem instability argued against reflexive attribution to acute coronary syndrome. Interpreting troponin as one data point within the overall clinical picture helped avoid unnecessary escalation while maintaining vigilance for evolving ischemia [12].

Dialysis readiness amid medical fragility

With an eGFR below 10 mL/min/1.73 m^2^ and persistent metabolic instability, dialysis planning became increasingly relevant. However, initiation decisions in medically fragile patients are rarely based on laboratory thresholds alone [13]. Creation of an AV fistula provided access readiness while allowing continued clinical reassessment, acknowledging the patient’s comorbid cardiopulmonary disease and recurrent infections, which could influence the timing and tolerance of renal replacement therapy [14].

Limitations

As a single-patient case report, these findings reflect one individual’s complex clinical presentation of ESRD, cardiopulmonary disease, and recurrent encephalopathy and should not be generalized to patients with isolated conditions. The multifactorial nature of this case limits our ability to definitively determine the relative contribution of metabolic, respiratory, hematologic, and infectious processes to the patient’s AMS. Diagnostic uncertainty was influenced by the limited availability of laboratory and radiographic data. Although a low serum ammonia level reduces the likelihood of hepatic encephalopathy, it does not exclude alternative metabolic or toxic contributors. Similarly, the absence of electrocardiographic changes lowered suspicion for acute coronary syndrome, yet silent or atypical ischemia cannot be entirely excluded. The lack of detailed baseline neurocognitive testing further restricts objective quantification of encephalopathy severity.

Dialysis-related decision-making was guided primarily by clinical judgment rather than strictly standardized criteria, which may limit reproducibility. Additionally, the absence of long-term follow-up data constrains the characterization of sustained outcomes and the overall disease trajectory. These limitations underscore the inherent constraints of single-patient analyses and reinforce the importance of dynamic reassessment in patients with ESRD and multiple comorbidities rather than anchoring to a single explanatory framework.

## Conclusions

In a medically complex patient with ADPKD-associated ESRD, HFpEF, and SPS, recurrent encephalopathy and equivocal pulmonary findings required sustained diagnostic vigilance rather than reliance on a single unifying explanation. This case emphasizes a pragmatic clinical approach in which encephalopathy was managed as a multifactorial syndrome and troponin elevations were interpreted within the broader clinical context rather than in isolation. Early preparation for renal replacement therapy through AV fistula creation facilitated readiness as metabolic instability and functional decline persisted, underscoring the importance of multidisciplinary care in patients with intersecting chronic disease processes.
